# On the Mode Employed for the Cure of the Venereal Disease, about the Middle and Latter Part of the Sixteenth Century

**Published:** 1818-12

**Authors:** 


*For the London Medical and Physical Journal.*

### *On the Mode employed for the Cure of the Venereal Disease, about the middle and latter Part of the Sixteenth Century.* Collected by EURYPHON.

Οὗτος μὲν πανάριστος, ὃς αὐτὸς πάντα νοήσῃ,
Ἐσθλὸς δ'αὖ κἀκεῖνος, ὃς εὖ εἰπόντι πίθηται.

THE subject of the present letter is the opinions of JULIUS PALMARIUS, respecting the means best adapted for the cure of the venereal disease. In the preface to his works,* he observes, that his motive for publishing the result of his experience, was to establish and extend the benefits to be

* De Morbis Contagiosis, 4to. Paris, 1578.

derived from the mode of treating syphilis, so successfully employed by his preceptor FERNELIUS, during a period of thirty years.

Fernelius, he however observes, would not, in general, undertake to cure the disease when it had become inveterate by long standing, or the ill effects of mercury; he only employed his remedies in recent cases. "But," he continues, "there are no cases, however severe and of long continuance, which may not be cured by the means I shall hereafter describe, if judiciously employed, and provided the patient has sufficient strength to bear the effects of them. And I solemnly assure my readers, that I recommend no remedy of which I am not perfectly convinced of the efficacy, from twenty years' extensive practice; I promise nothing which will not be confirmed by experience."

Although the method of Palmarius is similar in its principles and application to that of Fernelius, yet it may be useful to give a general account of it, as it will tend to enforce those points on which its success depends.

The patient, during the winter season, was confined to a room, into which great care was taken to prevent the entrance of currents of cold air, and it was kept warm by constant fires. On the first day, a dose of purgative medicine was given; on the following morning, some blood was taken from the arm, unless there were any thing present by which it was particularly contra-indicated. The purgatives were, in general, repeated every other day during the space of a week. The use of the strong decoction of guaiacum was then commenced with, which was given in the same way as directed by Fernelius. Cough, shortness of breathing, and pains in the back and limbs, not unfrequently made their appearance after a short time, which often alarmed the inexperienced physician, and furnished an argument for the calumny which the "tonscres" threw on the favourers of this plan. A little relaxation in the use of the remedy was all that was necessary; they then subsided in a few days. After the tenth or twelfth day, they began to join the use of the *amulet* with that of the guaiacum. The *amulet* of Palmarius is a preparation varying but little from the *opiate* of Fernelius. This was continued during a fortnight or three weeks, or longer in difficult cases. The patient, at the same time, took four ounces of the *cordial water** night and

* Thus prepared:—Take of sage-leaves, mint, marjorum, rue, hyssop, origanum, and betony, of each a handful; to be placed in an earthen vessel, and well covered with carduus benedictus water, and exposed to the heat of the sun for several days; the liquor is morning, after the subsidence of the sweating-fit. From twenty to thirty days was the time generally required to effect the cure. No wine was allowed during this time. After which, a weaker decoction of guaiacum was given for two or three weeks longer. These means, if persevered in for a certain length of time, were sufficient for the cure in almost every instance. But it might be more quickly and certainly effected by the use of the sweating-rooms, which were much employed in Germany. These rooms were heated by steam, imbued with the volatile parts of aromatic herbs: the patient sat in them as long as his strength would permit, night and morning, or only once a day if he were weak and delicate. After this, he lay down in bed for a short time.

The *diet* of the patients formed a very important part of the curative measures. Food was usually taken about four hours after the decoction of guaiacum. The quantity allowed was only barely sufficient to support the strength. In many cases, the guaiacum alone was nearly sufficient for this purpose; which Fracastorius has also expressed in his curious and elegant poem, *De Xyphilide.*

> "Quid mirandum æque memorem supra omnia victum,

> Quam tenuem quam magna cibi jejunia poscant?

> Quippe solet satis esse ipsum dum corpus alatur,

> Dum superet vita, et tantum ne membra fatiscant:

> Ne tamen, ah, ne tanta time, SACER illicet haustus,

> Ille modo ambrosiæ vires reficitque fovetque,

> Inque occulta gerit jejunis pabula membris."

Palmarius remarks, that, as sudden changes in diet are sometimes productive of unpleasant effects, he usually diminished the quantity of food in a gradual manner. A small quantity of roast meat was the utmost that was at length allowed; all soups and gravies were interdicted. Many took

---

then to be expressed, and the following ingredients added to it:—plantain leaves, balm, vervain, St. John's wort, lesser centaury, and pimpinella, of each two handfuls; macerate for four days; then add, scordium, devil's-bite, fennel, stone-parsley, bugloss, and borage, of each one handful; macerate and express. Then take—angelica, dittany, tormentil, betony, zedoary, à ℥ſs; cypress leaves, ʒiij; nutmegs, cloves, à ʒj; fennel-seeds, sorrel, carduus benedictus, juniper berries, à ʒij; ivory-shavings, hartshorn, aloes-wood, yellow sanders-wood, cinnamon, à ʒj; saffron, ℥ſs; to be made into a powder, and added to the above liquor, with half-a-pound of the *opiate*, the same quantity of mithridate, and four ounces of theriaca: these are to be macerated for six or eight days, and then distilled.

no food after noon. Some surgeons restricted the diet of their patients to two ounces of biscuit night and morning, with a few raisins and prunes. But Palmarius observes, that he could cure it with the guaiacum, opiate, &c. without reducing the system to an extreme degree of emaciation. "But," he remarks, "the diet should be as spare as the strength of the patient will possibly permit: the more closely this principle is followed, the more quickly and perfectly will the cure be effected: only 'cavendum est, ne quid nimis,'"

It is of great importance, he observes, that the mind of the patient should be kept free from all anxiety: amusements of the most exhilirating kind, as music, singing, agreeable conversation, and the presence of his friends, should be permitted.

There are two sections of the work of Palmarius, devoted to the consideration of the disease when complicated with other disorders, and that state of it which is the consequence of its being long neglected, or exasperated by the use of mercury. The general mode of treatment was the same, in these cases, with that which we have described; only the means were continued for a greater length of time, and great care taken to remedy the accidental derangement of the system.

The causes which induce the return of the disease are next considered, and the mode of treatment for it under these circumstances. Palmarius here endeavours to inculcate a knowledge of the advantages the method of cure he has described possesses over that by mercury, not only in its being more certain, perfect, and mild; but also because that, even in those cases in which relapses took place, the disease appeared in so much more mild a form than when it occurred after mercury had been employed.

Relapses after the mode of treatment above described, he observes, very rarely take place, except from the ignorance of the surgeon, or the refractory disposition of the patient. Many of them, as soon as the symptoms have disappeared, think they are cured, and cannot be persuaded to continue the use of the remedies. The disease, in these cases, generally appears on the skin; sometimes it affects the throat. Relapses after the use of mercury appear in diseases of the bones. In the former instances, they take place within three months after the remedies have been discontinued; in the latter, the disease may not be apparent for a much greater length of time: patients will have a fair outside, but the malady will be preying on the deep-seated parts. Relapses after the treatment by guaiacum, &c. are readily cured by a repetition of the same means; but those that take place after the use of mercury are cured with great difficulty.

Although my professed intention, in these letters, is merely to detail the opinions and observations of the authors to whom they allude, yet I cannot here refrain from making one reflection. The extreme spare diet, the copious use of guaiacum and other stimulating herbs, and the heated atmosphere in which the patients lived, were, when combined, powerful means for effecting rapid and extensive mutation of the system, and, from the excitement thus constantly kept up, of preventing the intervention of any other morbid action. —What are the effects of mercury, when exhibited in the usual mode for the cure of the venereal disease?

I shall resume this subject at a future period.

*London; Nov.* 1, 1818.

